# Live Attenuated Vaccines against Tuberculosis: Targeting the Disruption of Genes Encoding the Secretory Proteins of Mycobacteria

**DOI:** 10.3390/vaccines12050530

**Published:** 2024-05-12

**Authors:** Raja Veerapandian, Shrikanth S. Gadad, Chinnaswamy Jagannath, Subramanian Dhandayuthapani

**Affiliations:** 1 Center of Emphasis in Infectious Diseases, Department of Molecular and Translational Medicine, Paul L. Foster School of Medicine, Texas Tech University Health Sciences Center El Paso, El Paso, TX 79905, USA; 2 Center of Emphasis in Cancer, Department of Molecular and Translational Medicine, Paul L. Foster School of Medicine, Texas Tech University Health Sciences Center El Paso, El Paso, TX 79905, USA; 3 Department of Pathology and Genomic Medicine, Houston Methodist Research Institute & Weill Cornell Medical College, Houston, TX 77030, USA

**Keywords:** tuberculosis, live attenuated vaccines (LAVs), gene knockout, *Mycobacterium tuberculosis*, BCG, secretory antigens

## Abstract

Tuberculosis (TB), a chronic infectious disease affecting humans, causes over 1.3 million deaths per year throughout the world. The current preventive vaccine BCG provides protection against childhood TB, but it fails to protect against pulmonary TB. Multiple candidates have been evaluated to either replace or boost the efficacy of the BCG vaccine, including subunit protein, DNA, virus vector-based vaccines, etc., most of which provide only short-term immunity. Several live attenuated vaccines derived from *Mycobacterium tuberculosis* (*Mtb*) and BCG have also been developed to induce long-term immunity. Since *Mtb* mediates its virulence through multiple secreted proteins, these proteins have been targeted to produce attenuated but immunogenic vaccines. In this review, we discuss the characteristics and prospects of live attenuated vaccines generated by targeting the disruption of the genes encoding secretory mycobacterial proteins.

## 1. Introduction

Tuberculosis (TB) is caused by a Gram-positive bacterial pathogen, *Mycobacterium tuberculosis* (*Mtb*), which is known for its thick cell wall made up of long-chain fatty acids called mycolic acids [1]. Despite effective control measures, neither the death rate nor the incidence rate of TB has shown any sign of decline in recent years, and they remain at 1.3 million and 10.6 million in 2022 [2]. In addition, a quarter of the world population is estimated to have latent TB infection (LTBI) [3]. This situation is further aggravated by the emergence of multidrug-resistant tuberculosis (MDR-TB) and extensively drug-resistant tuberculosis (XDR-TB) strains, which require second-line drugs and prolonged care. In addition, HIV-TB coinfection contributes to about 12.8% of the total deaths due to TB, because HIV-1 depletes the CD4^+^ T cells associated with Th1 immune response [2]. The goal of the World Health Organization (WHO) is to reduce the number of new TB cases by 90% in the year 2035, and achieving this goal requires the development of novel drugs and therapeutic agents for the treatment of the disease and, importantly, efficacious preventive and therapeutic vaccines.

Currently, Bacille Calmette–Guerin (BCG) is the only approved vaccine for TB. It was derived from the virulent *Mycobacterium bovis*, the pathogen that causes TB in cattle, when it was passaged in culture media over two hundred times [4,5]. An estimated four billion doses of BCG have been administered to infants with very few adverse incidences, indicating that it is the safest vaccine available [6]. However, a major weakness of BCG is its variable efficacy (0–80%) in different populations of different ethnicities [5,7]. Several reasons account for this discrepancy, including (a) the geographical location of the populations tested [8], (b) environmental mycobacteria that share antigens with BCG [7], and (c) variation in the antigenic profile of BCG sub-strains due to genetic differences [9]. In addition, it has been proposed that humans exposed to atypical mycobacteria [10] and helminthic infections will have increased Th2 response, which can also reduce the efficacy of BCG vaccination [11,12]. Regardless of these factors, the consensus is that BCG prevents TB meningitis in children but has no effect on the most common pulmonary TB in children and adults [5,7]. A recent meta-review also reiterates that infant vaccination using BCG can prevent tuberculosis in young children but is ineffective in adolescents and adults [13]. This underscores the need for a better primary vaccine and a booster that can induce BCG-induced primary immunity.

The past two decades have witnessed the development of many vaccines against TB, and some of these vaccines have advanced to clinical trials [14]. These include platforms based on protein subunits, viral vectors, recombinant mycobacterial live attenuated vaccines (LAVs), killed whole cell vaccines, and an mRNA-based vaccine [6,15,16,17,18,19,20,21]. Compared to other vaccines that transiently express antigens, LAVs are considered superior to others because they tend to persist for extended periods, encoding antigens and enabling the longer-lasting stimulation of immune cells to induce protective immune responses [22,23]. Further, the antigens produced by LAVs are closer to native antigens, which are properly folded proteins, carbohydrates, and lipids [24,25]. Moreover, these diverse antigens will likely stimulate multiple immune cell populations like subsets of T and B cells and phenotypes such as NK and innate T cells. Another advantage of using LAVs is that their cell wall components can stimulate innate immunity [26,27]. Thus, cell wall lipids such as trehalose dimycolate (TDM) can activate Mincle receptor-inducing trained immunity [28,29], whereas other glycolipids and several pathogen-associated molecular patterns (PAMPs) can trigger pattern recognition receptors (PRR) like TLR-2 and TLR-4 [30]. However, great caution should be exercised when considering lipids as inducers of the immune response because some essential *Mtb* lipids, like sulfoglycolipids, inhibit the innate immune response [31]. More importantly, the loss of phthiocerol dimycocerosates (PDIMs) and phenolic glycolipids (PGLs) in the BCG Pasteur strain reduces the efficacy of the BCG vaccine, highlighting the importance of PDIMs/PGLs [32]. Another attractive strategy for modifying live attenuated vaccines (LAVs) is the addition of major *Mtb* proteins to BCG to create recombinant BCG vaccines for better efficacy. However, our review is focused explicitly on *Mtb* or BCG knockouts as LAVs.

**Figure 1:**
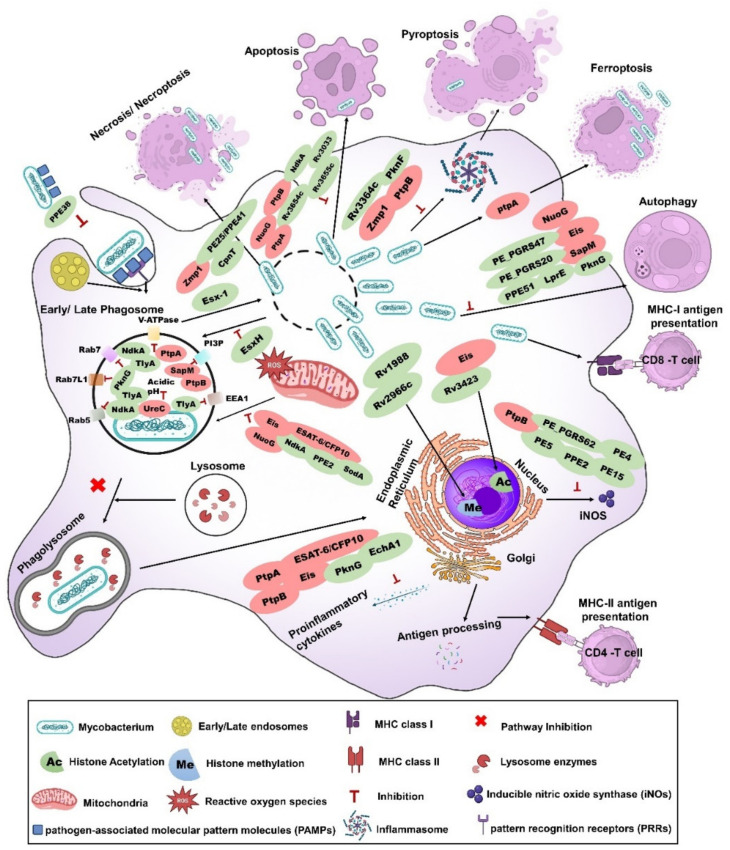
**Subversion of phagocytosis and related processes by mycobacterial secreted proteins. Phagosome maturation**: *p*attern recognition receptors (PRRs) of macrophages recognize mycobacteria through mycobacterial pathogen-associated molecular patterns (PAMPs), resulting in the engulfment of bacilli by a phagosome, an organelle derived from the plasma membrane. Phagosomes undergo a series of steps called phagosome maturation to digest the engulfed bacilli and present the antigens to the immune cells. Additionally, phagosomes fuse with early endosomes, late endosomes, and, subsequently, with lysosomes to acquire the materials/properties required for the killing/digestion of the pathogen. However, intracellular mycobacteria like *M. tuberculosis* (*Mtb*), *M. bovis, M. marinum,* and BCG have multiple strategies to protect them against the phagocytic processes. *M. marinum* secretes PPE38 [65] to block the phagocytosis of the bacilli. *Mtb* secretes several secretory proteins to inhibit the phagosome maturation process. SapM [34] and PtpB [66] dephosphorylate phosphatidylinositol-3-phosphate (PI3P) to inhibit phagosome maturation, whereas PtpA protein inhibits phagosome acidification by blocking Vacuolar-type ATPase (V-ATPase) [67]. TlyA inhibits Early Endosomal Antigen-1 (EEA1), Ras-related protein 5 (RAB5), and RAB7 recruitment [68]. NdkA inhibits RAB5 and RAB7 [69], while PknG inhibits RAB7L1 [70]. Additionally, *Mtb* secretes UreC to alkalize the phagosomes [71]. In addition, *Mtb* perforates the phagosome and escapes to the cytosol using the concerted action of phthiocerol dimycocerosates (PDIM) and ESX-1 system [72]. Host efforts to repair phagosomal rupture were blocked by the EsxH protein [59]. **ROS/iNOS:** Host NADPH oxidases (NOX) from the cytoplasm and mitochondrial electron transport chain are the primary sources of reactive oxygen species (ROS) production. ROS is blocked by *Mtb* proteins like Eis, ESAT-6/CFP-10, NuoG, NdkA, PPE2, SodA [73,74,75,76], and inducible nitric oxide synthase (iNOS), while the mediated production of NO is blocked by PtpB, PPE2, PE_PGRS62, PE5, PE15, and PE4 [77,78,79,80]. **Epigenetic regulators:** *Mtb* secretes proteins like Rv1988 [81] and Rv2966c [82] to methylate host DNA and proteins like Eis [83] and Rv3423.1 [84] to acetylate host DNA to manipulate the host immune response. **Proinflammatory cytokines:** *Mtb* secretes proteins like PtpA, PtpB, ESAT-6/CFP-10, Eis, EchA1, and PknG [77,85,86,87,88,89] to inhibit proinflammatory cytokines. **Cell death pathways:** Cytosolic escape of the pathogen leads to activation of various cell death pathways like necrosis mediated by Zmp1, CpnT, and PE25:PPE41 [90,91,92] or ferroptosis via PtpA which benefits the pathogen [93]. The cytosolic presence of bacilli DNA or RNA triggers various pathways like apoptosis, autophagy/xenophagy, and pyroptosis, which are detrimental to the pathogen. *Mtb* secretes NuoG, PtpA, PtpB, NdkA, Rv3654c, Rv3655c, and Rv3033 to block apoptosis [36,75,77,94,95,96]. Proteins like Zmp1, PknF, PtpB, and Rv3364c block inflammasome activation and/or pyroptosis [35,97,98,99]. Autophagy/xenophagy pathways are blocked by proteins like NuoG, Eis, SapM, PE_PGRS20, PE_PGRS47, PPE51, LprE, and PknG [86,100,101,102,103,104,105]; however, these proteins block autophagy indirectly by blocking early/late phagosome proteins. **Note**: Some live attenuated *Mtb* or BCG vaccines described in this review lack one or more secretory proteins mentioned above, and they are designated in this figure with rose-colored oval shapes.

**Figure 2:**
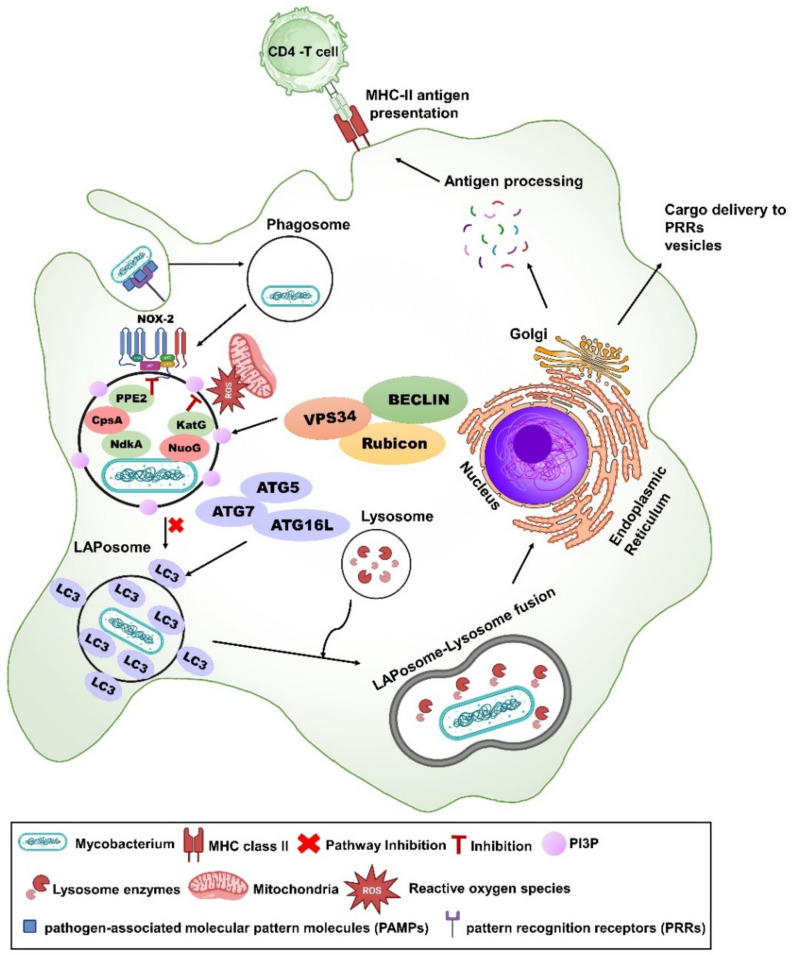
**Subversion of LC3-associated phagocytosis (LAP) by secreted proteins of mycobacteria.** LC3-associated phagocytosis is initiated by macrophage via engaging specific receptors like TLR1/2, TLR2/6, TLR4, Fc receptors, CLEC7A/Dectin-1, and TIM4, and it also recognizes apoptotic, necrotic, and entotic cells [124]. Once the bacilli are engulfed within a single membraned phagosome, phosphatidylinositol-3-phosphate (PI3P) is recruited over the phagosome, which is generated by the PtdIns3K (Class lll phosphatidylinositol 3-kinase) complex. LAP and canonical autophagy share common features and unique features in their pathway. Both require PI(3)P production and common machinery like BECLIN, VPS 34, ATG5, ATG7, ATG16L, TSG101, and RAB7 to recruit LC3 over the phagosome [125]. Recruitment of LC3 over a single-layered membrane is called LAPosome. LAPosome subsequently fuses with lysosome to eliminate *M. tuberculosis* (*Mtb*). Some unique proteins involved in the LAP pathway are Rac, p47, p40, p67, p22, gp91phox, and Rubicon [125]. However, *Mtb* secretes multiple proteins to block the LAP pathway. *Mtb* proteins like CpsA, NdkA, and PPE2 inhibit the phagosomal recruitment of NADPH oxidase (NOX-2 complex) to the phagosome, whereas NuoG and KatG proteins neutralize reactive oxygen species (ROS) [126]. **Note**: some live attenuated *Mtb* or BCG vaccines described in this review lack one or more secretory proteins mentioned above, and they are designated in this figure with rose-colored oval shapes.

**Figure 3:**
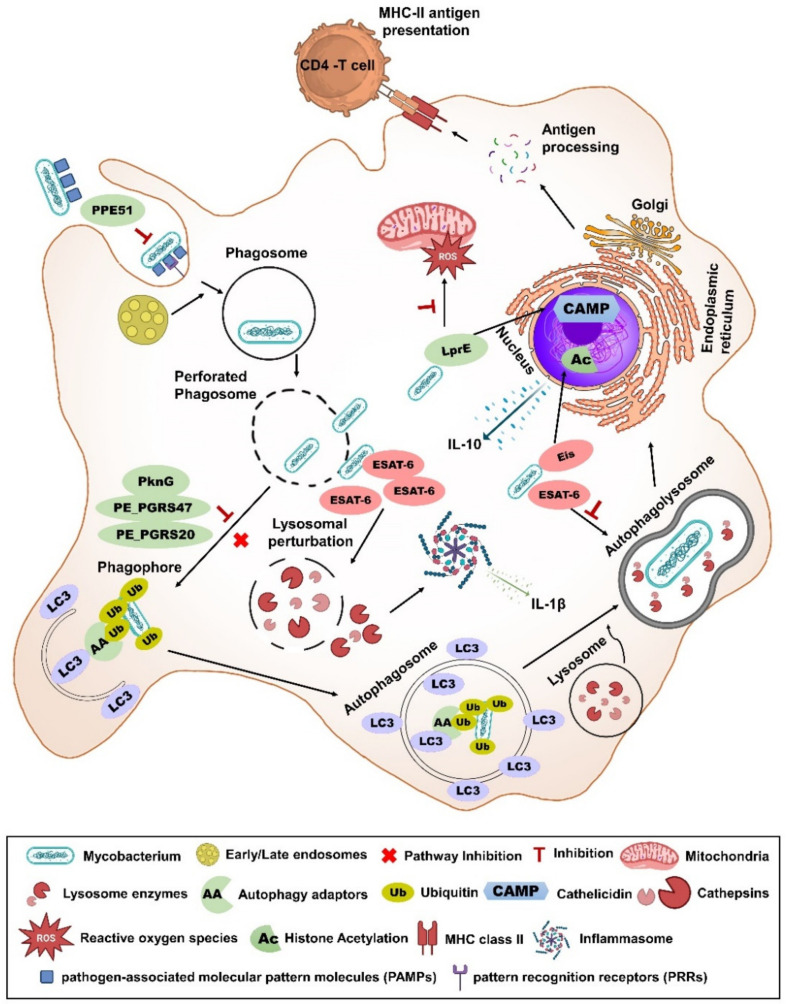
**Subversion of xenophagy/autophagy by secreted proteins of mycobacteria.** *M. tuberculosis* (*Mtb*) is well-known for inhibiting the maturation of phagosomes and their subsequent fusion with lysosomes. Through the ESX-1 secretion system and the cell envelope lipid PDIM, *Mtb* perforates the phagosome membrane and escapes to the cytosol. The first defensive step of a host is initiating the autophagy pathway by successfully binding ubiquitin to bacteria, followed by the recruitment of autophagy adaptors such as p62, OPTN, TAX18P1, NBR1, and TOLLIP. Subsequently, these autophagy adaptors engross with microtubule-associated-protein-1 light chain 3 (LC3) to deliver *Mtb* to autophagosomes. However, *Mtb* has multiple evasion strategies to escape from the host autophagic pathway via the secretion of various protein effectors. *Mtb* SapM inhibits Rab7, a late endosome marker required for autophagosomes to fuse with the lysosomes [100]. *Mtb* protein Eis inhibits autophagy via suppression of c-Jun N-terminal kinase (JNK)-mediated reactive oxygen species (ROS) signaling [86] or through the acetylation of host histone, which upregulates IL-10 and activates the Akt/mTOR/p70S6K pathway [83]. *Mtb* secretes LprE to suppress autophagy by inhibiting the expression of cathelicidin antimicrobial peptide (CAMP) via the p38 MAPK pathway [104]. *Mtb* protein PE_PGRS47 suppresses autophagy by inhibiting LC3 colocalization [171]. PknG blocks Rab14 to inhibit the autophagosome maturation [103]. PE_PGRS20 and PE_PGRS47 inhibit autophagy by interacting with RAB1A, which recruits the ULK1 (unc-51-like autophagy activating kinase 1) complex to the pre-autophagosome [102]. PPE51 inhibits autophagy by blocking the activation of the extracellular signal-regulated kinase 1/2 (ERK1/2) pathway [105]. ESAT-6 released by *Mtb* perforates the lysosomes and autophagosomes, leading to perturbation of membranes. Damage to membrane releases cathepsin B in the cytosol and causes the subsequent activation of NLRP3-inflammasome and release of matured IL-1β [172]. Secreted ESAT-6 also affects autophagy flux in dendritic cells [173]. **Note**: Some live attenuated *Mtb* or BCG vaccines described in this review lack one or more secretory proteins mentioned above, and they are designated in this figure with rose-colored oval shapes.

A major approach to derive LAVs against TB depends on a ‘rational deletion of genes’ in the chromosomes of *Mtb* and BCG [33]. Often, the target genes play a critical role in immune evasion by *Mtb*. Importantly, there is a strong link between immune evasion mechanisms and the ‘secretory proteins’ of mycobacteria, because many of these seem to be released into the host environment to modulate phagolysosomal (PL) fusion, autophagy, apoptosis, the modulation of cytokines, the intracellular survival of pathogens, and other related antimicrobial pathways [34,35,36,37] (Figure 1, Figure 2 and Figure 3). Coincidentally, many LAVs for TB are based on deleting genes encoding ‘secretory proteins’ or their regulators and transporters (Table 1 and Table 2). In this review, we describe the immunological parameters of LAVs deficient in secreted protein(s) and their efficacy compared with the BCG vaccine, which also secretes several antigenic proteins [38]. We specifically address LAVs deficient in secretory antigens, since other gene knockout mutants have been described by others [15,18,39,40].

## 2. Secretory Systems of Mycobacteria

To determine the significance of host immune modulation by *Mtb*, it is imperative to understand the secretory systems of mycobacteria. Bacteria generally have intriguing mechanisms to transport some of their proteins across the cytoplasmic membrane, which are called secretion systems or secretory pathways [41]. They transport proteins that need to be localized in the periplasm, outer membrane, or surface or released in the extracellular environment and into the host cells. The two major secretory pathways in bacteria are the general secretory pathway (or Sec pathway) and the twin-arginine translocation pathway or TAT pathway [41]. Both are highly conserved systems and are present in most bacteria [42,43]. In addition to Sec and TAT pathways, Gram-negative pathogenic bacteria have special pathways to transport virulence factors. There are six such special pathways, which are named type I through type VI secretory systems. Some of these systems, like the type III secretion system in *Salmonella* species, form microneedles to inject the effector proteins directly into the host cells [41]. The Gram-positive mycobacteria were initially thought to have only the Sec and TAT pathways to secrete proteins. However, a new special secretory system was later identified in mycobacteria [44] and was named the type VII secretion system [45], in line with the previously labeled secretion systems.

In contrast to other species, mycobacteria have two Sec pathways, SecA1 and SecA2 [44]. While transporting proteins through SecA1 requires a signal sequence, no signal sequence is required for transporting proteins through SecA2. The SecA2 pathway in *Mtb* appears to mainly transport proteins related to pathogenesis, such as SodA, SapM, and PknG [46]. On the other hand, the TAT pathway requires a TAT signal sequence to transport proteins [41]. In *Mtb*, the TAT pathway transports fewer proteins associated with pathogenesis, including phospholipase A and C [46].

Further, the Type VII secretion system in *Mtb* and related mycobacteria has five export systems, and they are named ESX-1 through ESX-5 [47,48,49]. Each system has a cluster of genes to encode the proteins, facilitated by the structural proteins required for transportation. The ESX systems recognize the substrates or the proteins to be transported by the YxxxD/E signal (amino acid) motif in their sequence [50]. Among the five ESX systems, ESX-4 seems to be the oldest system, and others appear to have evolved through duplication events [51,52]. Notably, the gene cluster for this system lacks genes encoding PE/PPE proteins, although the other four systems do have genes to encode these proteins.

Nonetheless, the ESX-1 system seems to be the most well-studied and is responsible for the secretion of EsxA (ESAT-6) and EsxB (CFP-10) proteins in *Mtb* and related pathogens [53,54,55,56]. These proteins are both virulence factors and immunodominant antigens, and coincidentally, their absence leads to an avirulent phenotype in *Mtb* [55]. ESAT-6 enables the bacteria to lyse the phagosomal membrane with the aid of the chaperone CFP-10, a strategy that is missing in BCG vaccine strains [57]. The ESX-1 system also constitutes the Region of Difference 1 (RD1), deleted in the BCG vaccine strains [54]. Indeed, the attenuation of BCG appears to be due to the absence of the RD1 region in its chromosome, because the complementation of BCG with the RD1 region restores its virulence [54]. Both ESX-1 and ESX-2 systems are intact in *Mtb* and other related pathogens, but little information is available about its secreted products and role in pathogenesis [58].

Further, ESX-3 and ESX-5 systems participate in the secretion of their proteins, and the latter plays a significant role in immune modulation or inflammasome activation [59,60,61,62,63]. ESX-4 is different in functionality, and unlike other systems, it plays a vital role in conjugation between bacteria [64]. Overall, mycobacteria use Sec, TAT, and ESX systems to transport proteins that regulate virulence and immunogenicity. However, some secreted proteins do not seem to have specific export signatures.

## 3. Mycobacterial Vaccines Deficient in Secreted Protein(s)

As noted above, the preference for LAVs over others is due to their superior number of antigens and ability to stimulate immune response for a more prolonged period in vivo [22]. BCG, an excellent example of an LAV, is poorly effective in adults, requiring a replacement or a booster. Initial studies to improve BCG through the recombinant overexpression of antigenic genes did improve its efficacy against TB in the mouse model [106,107,108,109,110]. In particular rBCG30 and BCG85B vaccines showed remarkable efficacy against TB in mice but did not advance to clinical trials. One possible reason is that they lacked the immunogenic RD1 region that encodes ESAT6 and CFP10, which are paradoxically related to virulence in *Mtb*. An extensively explored approach is deleting genes in BCG and *Mtb* to derive LAVs. In the past two decades, over fifty mycobacterial mutant strains with deletion or disrupted gene(s) in the chromosome have been tested for their vaccine efficacy in animal models, regardless of their attenuation status [18,110]. Interestingly, a large proportion of the LAVs tested so far are those that lack secretory proteins (Table 1 and Table 2). The primary candidate LAVs lacking secretory proteins are discussed below.

### 3.1. Ag85 Complex

*Mtb* expresses three secreted fibronectin proteins (Fbp), namely FbpA (Rv3804), FbpB (Rv1886c), and FbpC (Rv0129c) [111,112]. All three of them are major antigens (Ag), and hence, they are also known as Ag85A (31 kDa), Ag85B (30 kDa), and Ag85C (31.5 kDa) and collectively as the Ag85 complex of proteins [111,112]. The amino acid sequences of these proteins are highly conserved among mycobacterial species and, in particular, the *Mtb* complex. Besides fibronectin-binding activity, these proteins show mycolyl transferase activity essential in assembling the mycobacterial cell walls [113]. Armitige et al. [114] disrupted the genes *fbpA* and *fbpB* in *Mtb* and assessed the mutant strains (Δ*fbpA* and Δ*fbpB*) for their growth and survival in culture, macrophages, and mice [114,115]. The Δ*fbpA* strain was later shown to protect C57BL/6 mice against challenges with an efficacy similar to BCG [115]. To our knowledge, this is the first study demonstrating that an *Mtb* mutant lacking a secretory protein antigen is effective as a vaccine against TB. The Δ*fbpA* candidate vaccine induced high levels of phagosome maturation, proinflammatory cytokines, and Th1 immune response and an increased expansion of CD4+ CXCR3+ IFN-γ+ cells in mice after vaccination [116]. Although recombinant mycobacterial strains over-expressing Ag85B (FbpB) and Ag85C (FbpC) also protect mice against TB [106,117], thus far, *Mtb* mutants ‘lacking’ these proteins have not been tested for vaccine efficacy. Notably, a BCG strain lacking the Ag85B protein showed efficacy against TB in mice, like BCG [118]. This is not surprising, because BCG naturally has a mutation in Ag85B, which affects the mycolyl transferase activity and its stability [119]. It is intriguing to note that Ag85 complex proteins are immunogenic in mice, guinea pigs, and human immune cells, and individual deletion yields mutants defective in cell wall lipids due to their mycology transferase activity. The Δ*fbpA* has reduced levels of trehalose dimycolate (TDM) in its cell wall [120] and TDM has been thought to be a virulence factor [121]. Therefore, we are pursuing the hypothesis that the selective deletion of Ag85 complex genes in combination with other functionally characterized genes will lead to markedly immunogenic and concurrently attenuated vaccines [122] (under submission). It should be noted that the MTBVAC vaccine, which is currently undergoing clinical trials, shows a higher secretion of Ag85 proteins, indicating its role in inducing an immune response [123].

### 3.2. LpqH

Proteomics identified the LpqH (Rv3763) protein in the cell wall and the culture filtrates of *Mtb*. LpqH is a 19 kDa lipoprotein that plays multiple roles in the virulence of *Mtb*, such as TLR-2 interaction, the induction of humoral and T cell-mediated responses, and apoptosis [127,128]. Macrophages infected with a mutant strain of *Mtb* lacking this protein (Δ*19*) show a reduced surface expression of MHC-II molecules and the secretion of cytokines IL-1β, IL12p40, and TNF-α [129]. The Δ*19* strain was highly attenuated for growth in C57BL/6 mice compared to wild-type *Mtb* H37Rv. Despite poor in vivo growth, in mice, LpqH elicited a protective efficacy equal to BCG (1 log^10^ reduction in *Mtb* counts in lungs) [130]. Reduced *Mtb* growth correlated with increased IFN-γ-secreting CD4+ and CD8+ T cells comparable to mice receiving the BCG vaccine, although the lung granulomas of Δ*19* strain-vaccinated mice had more lymphocytes than BCG-vaccinated mice, which had more vacuolated foamy macrophages.

### 3.3. LprG

This is another mycobacterial lipoprotein agonist of TLR-2 receptors in macrophages [131], which, in combination with immunomodulatory lipids, seems to prevent PL fusion in macrophages and limit antigen presentation through the MHC-II pathway [132]. The *Rv1411c* gene encoding LprG is transcriptionally linked to *Rv1410c*, which encodes a transmembrane efflux pump, and the deletion of the *1411c-1410c* locus in *Mtb* (*ΔlprG*) attenuates its growth in immunodeficient mice [133]. The growth attenuation appears to be due to the accumulation of intracellular triacylglycerides (TAG) and altered bacterial metabolism [133]. The Δ*lprG* mutant was evaluated for its immunogenicity and vaccine efficacy in three mouse strains [134]. While Δ*lprG*-induced protection was comparable to BCG in C57BL/6 and BALB/c mice, it showed a 0.9 log^10^ better decrease in *Mtb* load in the lungs of C3HeB/FeJ mice; the latter developed necrotizing TB granulomas, similar to humans [134]. Variable protection was also reflected in pathology as *ΔlprG*-vaccinated mice showed fewer granulomas than mice given BCG. Further, compared to BCG, increased Ag-specific CD4+ positive T cell responses, lower percentages of PD-1 positive T cells, and increased antigen-specific IL-17A-secreting T cells were found in the lungs of Δ*lprG*-immunized mice [134]. These data were confirmed by a recent study where increased protection was observed in Δ*lprG*-immunized C3HeB/FeJ mice given a low dose aerosol infection. Protection correlated with elevated serum levels of IL-17A, IL-6, CXCL2, CCL2, IFN-γ, and CXCL1 [135]. The Δ*lprG* mutant illustrates that LAVs may show variable protection in mouse strains of different genetic backgrounds.

### 3.4. BfrB

Iron limitation is a major factor affecting host–pathogen interactions [136]. Though *Mtb* has multiple mechanisms to sequester iron from the host, it uses bacterioferritin (BfrB, Rv3841), a secretory protein [137,138], to store the iron when it is abundant and to release it when required. A Δ*bfrB* mutant of *Mtb* could not establish a chronic infection in mice [139]. When it was used as a vaccine against *Mtb* H37Rv in mice, the mutant generated protection comparable to BCG, although they differed in organ pathology and lung transcriptomic signatures [140]. Further, at eight weeks post-vaccination using Δ*bfrB*, mice had reduced inflammation, smaller lung granulomas, and extensive fibrosis [140].

### 3.5. CpsA

Encoded by the gene *Rv3484*, CpsA belongs to the LytR-CpsA-Psr family of conserved proteins related to cell wall assembly in Gram-positive bacteria [141]. Koster et al. reported that *Mtb* lacking the CpsA protein (Δ*cpsA*) enhances LC3-associated phagocytosis (LAP) and the recruitment of NADPH oxidase to their phagosomes, suggesting that secreted CpsA protein inhibits these innate immune responses to survive inside the host [125]. In addition, the Δ*cpsA* strain showed defective growth in mice, which was restored by complementation using a functional *cpsA* gene [125]. Since LAP can increase antigen presentation through the MHC-II pathway, the authors proposed that the Δ*cpsA* could be a potential vaccine candidate and created a new mutant *mc^2^6206*Δ*cpsA* by deleting *cpsA* in *mc^2^6206*, which is an auxotrophic mutant strain (Δ*leuCD* and Δ*panCD*) [142]. Despite increased LC3 trafficking in BMDMs, *mc^2^6206*Δ*cpsA* showed protection similar to BCG in C57BL/6 mice challenged with *Mtb* H37Rv [142]. Given its ability to increase LC3 trafficking, it is unclear why there was no better protection than BCG, which uses a sapM-dependent mechanism to evade autophagy. A deletion of *cpsA* in *Mtb* may generate a more protective phenotype.

### 3.6. BioA

The enzyme 7,8-diaminopelargonic acid synthase, also known as BioA (Rv1568), is one of the four enzymes associated with synthesizing biotin molecules in *Mtb* [143]. It appears to be critical for the acute and chronic infection of mice infected with *Mtb* [144]. *Mtb* Δ*bioA* was severely attenuated in guinea pigs regardless of aerosol or intradermal route of infection, and intriguingly, the lungs of Δ*bioA*-infected guinea pigs did not show live bacteria after six weeks post-infection [145]. Although vaccinating guinea pigs using this mutant significantly reduced the bacillary burden in the lungs and spleens, repeated vaccination before the *Mtb* challenge reduced its efficacy [145]. This study illustrates that the hyperattenuation of *Mtb* may not always correlate with vaccine efficacy.

### 3.7. Gln Proteins

Glutamine synthetase activity in the culture filtrate of *Mtb* has been reported [146]. The *Mtb* genome has multiple genes encoding glutamine synthetase, namely GlnA1 (Rv2220), GlnA2 (Rv2222c), GlnA3 (Rv1878), GlnA4 (Rv2860c), and a regulator protein GlnE (Rv2221c). However, only GlnA1, GlnA3, and GlnA4 were identified in the culture filtrate of *Mtb* using proteomics [138,147]. Lee et al. [148] characterized the GlnA1, GlnA2, GlnA3, and GlnA4 mutants and a triple mutant for GlnA1EA2. Of these, only *glnA1* and *glnA1EA2* were essential for the growth of *Mtb*, and they were auxotrophic to glutamine. Both Δ*glnA1* and Δ*glnA1EA2* showed attenuated growth in immunodeficient SCID mice and immunocompetent C57BL/6 mice [148]. Additional studies using C57BL/6 mice revealed that they protected mice like BCG [148]. The inability of these mutants to multiply in vivo appears to have discouraged their further validation. GlnA1 is abundant in mycobacterial extracellular vesicles (MEV), suggesting that it may serve as a diagnostic marker [149].

### 3.8. SapM

Among the three known secreted phosphatases (SapM, PtpA, and PtpB) of *Mtb*, SapM (Rv3310) is the first to be identified as an acid phosphatase [150]. After *Mtb* infects human macrophages, its survival within macrophages depends upon the inhibition of phagosome maturation. SapM is one of the key enzymes implicated in phagosomal maturation arrest during *Mtb*–macrophage interactions [46,151]. SapM dephosphorylates phagosomal membrane-bound phosphatidylinositol 3-phosphate (PI3P), a lipid molecule associated with the recruitment of downstream effector Rab proteins essential for phagosomal maturation, also called phagosome–lysosome (PL) fusion [34]. Essentially, the SapM-mediated removal of a phosphate molecule from PI3P affects its structure and reduces the recruitment of effector proteins, inhibiting PL fusion. Further, SapM also inhibits autophagy by blocking Rab7, a small GTPase required for lysosomal fusion [100]. Two independent studies used *Mtb*Δ*sapM* to define the role of SapM during PL fusion [152,153]. Upon the infection of macrophages, these mutants were seen more frequently in the matured phagolysosomal compartments enriched with lysosomal markers like LAMP1 when compared to macrophages infected with wild-type *Mtb* [152,153]. The *MtbΔsapM* mutant also showed attenuated growth in macrophages and in the lungs and spleen of guinea pigs [152,153]. Consistent with PL fusion competence and attenuation, the mutant showed increased in vitro and in vivo immunogenicity; its immunogenicity was further increased when *fbpA* was deleted, yielding an *Mtb*Δ*fbpA*-Δ*sapM* double knockout (DKO) mutant [152]. Notably, mice vaccinated with the DKO strain and challenged with *Mtb* showed better protection (>1 log^10^) than mice receiving the BCG vaccine [122]. Others reported similar results when guinea pigs were vaccinated using a triple knockout strain of *Mtb* (*Mtb*Δ*mms*) that lacked three phosphatases (PtpA, PtpB, and SapM) [154]. *Mtb*Δmms was more effective in decreasing *Mtb* CFUs in the lungs of guinea pigs (3.60 log10) compared to the lungs of guinea pigs receiving BCG vaccination (4.43 log10) [154]. These two studies suggest that the deletion of the same gene increases the vaccine efficacy in the KO mutants, even if the other deleted genes are functionally different, mainly because the deletion of the *bioA* gene in *Mtb*Δ*mms* (*Mtb*Δ*mmsb*) had no additional benefit [155]. It is relevant to recall here that a BCG *sapM* mutant (BCGΔ*sapM*) transposon mutant also shows enhanced protection as a vaccine against *Mtb* in BALB/c mice [156]. BCGΔ*sapM* enriched CD11c+MHC-II intCD40int dendritic cells (DCs) in the draining lymph nodes and was found to be safer than the BCG in SCID mice [157]. Since *sapM* deletion increased the vaccine efficacy of both *Mtb*- and BCG-derived mutants, it may be an essential target for more effective vaccines.

### 3.9. Ptp

PtpA (Rv2234) and PtpB (Rv0153c) are the only two secreted phosphotyrosine protein phosphatases (Ptp) identified in *Mtb* [158]. By interacting with host signaling partners, they can modulate the cellular pathways of the host cells [159]. The genes encoding PtpA and PtpB have been disrupted in the chromosome of *Mtb*, and their roles in pathogenicity are reported [160,161]. PtbA primarily affects phagosomal maturation by interacting with H subunit V-ATPase, an enzyme required for phagosomal lumen acidification, and PL fusion by dephosphorylating the vacuolar protein sorting-associated protein 33B (VPS33B), a late endosomal molecule [67,160]. In addition, PtpA plays a critical role in suppressing innate immune responses by interacting with ubiquitin, dephosphorylating JNK, and regulating host genes such as GADD45A [85,158]. Similarly, PtpB has been reported to promote the survival of *Mtb* H37Rv by suppressing iNOS, IL-1β, and IL-6 [77], thus suppressing innate immune responses through ERK1/2 and Akt pathways [162] and interacting with ubiquitin and inhibiting host cell pyroptosis [97]. Coincidentally, the Δ*ptpB* strain is attenuated for growth in macrophages and guinea pigs compared to wild-type *Mtb* [161]. Further, an *Mtb* strain with triple deletions (*ptpA*, *ptpB*, and *sapM*) protected against TB in a guinea pig challenge model better than BCG. [154]. Although the individual roles of Δ*ptpA* or Δ*ptpB* in contributing to vaccine efficacy remain unclear, the fact that they affect multiple host processes justifies their deletion as a strategy to derive vaccines.

### 3.10. Zmp1

Zinc-containing metalloprotease 1, or Zmp1, is encoded by the gene *Rv0198c*. Masters et al. [35] first generated a *zmp1* deletion mutant in *Mtb* and BCG, showing that it is crucial in preventing inflammasome activation in macrophages and phagosomal maturation. Macrophages infected with an *Mtb*Δ*zmp1* mutant not only secreted more IL-1β but also enhanced PL fusion, indicating that it is a key virulence factor [35]. In addition, Zmp1 causes necrotic cell death and the dissemination of *Mtb* [90]. Interestingly, the deletion of *zmp1* has similar effects in both *Mtb* and BCG fields [35]. This led Sanders et al. to evaluate *zmp1* deletion in the BCG vaccine [163,164]. Mouse bone marrow-derived dendritic cells (DCs) infected with BCG and BCGΔ*zmp1* were compared for their antigen presentation to T cells using *Mtb* Ag85A-specific MHC-II restricted hybridoma T cells [163]. As expected, DCs infected with BCGΔ*zmp1* displayed enhanced antigen presentation, suggesting that the BCGΔ*zmp1* strain is immunogenic [163]. BCGΔ*zmp1*-vaccinated mice showed a stronger delayed-type hypersensitivity (DTH) reaction, and splenocytes showed heightened IFN-γ levels in response to PPD stimulation when compared to splenocytes from BCG-immunized mice [163].

Further, BCG Pasteur lacking Zmp1 and BCG Denmark strain were compared with wild-type BCG Denmark (Danish) for efficacy against TB in guinea pigs [164]. Both mutants showed impressive protection against the *Mtb* challenge and reduced the lung *Mtb* burden by approximately 0.5 log^10^ CFU compared to BCG Denmark. This observation is remarkable because BCG Denmark, on its own, showed about 1.8 log^10^ CFU reduction compared to unvaccinated controls [164]. BCGΔ*zmp1* has a high safety profile in SCID mice, particularly the Danish BCGΔ*zmp1*, which is hyper-attenuated [164]. Although the mechanisms underlying protection are unclear, BCGΔ*zmp1* strain was about to enter into a phase I clinical trial in 2017 [21].

### 3.11. Eis

The enhanced intracellular survival (EIS) protein is a secretory protein of *Mtb* encoded by the gene *Rv2416c* [165]. The name EIS is because it enhances the survival of *M. smegmatis* within macrophages [166]. Paradoxically, an *eis* deletion mutant of *Mtb* (*Mtb*Δ*eis*) showed no defect in intracellular survival within macrophages but induced higher levels of proinflammatory cytokines [167]. Such macrophages also showed increased reactive oxygen species (ROS) generation, autophagy, and cell death [86]. The Eis protein is an enzyme with aminoglycoside N-acetyltransferase activity. Thus, it seems capable of modulating or inhibiting proinflammatory responses, JNK-dependent autophagy, ROS generation, and, to some extent, phagosome maturation by acetylating the host phosphatase protein DUSP16/MKP-7 [73]. The inhibition of autophagy by Eis also seems to be due to the acetylation of histone H3 (Ac-H3), which can upregulate the expression of IL-10 and, as a consequence, activate the Akt/mTOR/p70S6K pathway [83]. Eis is the first secreted protein of *Mtb* to epigenetically modify macrophages. Recently, its homolog from BCG (*BCG_2432c*) was knocked out in BCG (China sub-strain), and the mutant Δ*BCG_2432c* was tested as a vaccine against TB in a C57BL/6 mice [168]. Remarkably, Δ*BCG_2432c*-immunized mice showed approximately a 2.0 log^10^ reduction in CFU in the lungs compared to mice immunized with wild-type BCG (China sub-strain). This enhanced protection was likely due to elevated levels of IFN-γ+ CD4+ TEM (effector memory T cells) and IL2+CD4+TCM (Central memory T cells) in the lungs and spleens of Δ*BCG_2432c*-immunized mice. This is the first study demonstrating a significant reduction in *Mtb* CFU in mice by a BCG vaccine with a single gene deletion in the chromosome. Because of its effect in mice, the deletion of the *eis* gene in *Mtb* appears to be a promising approach for TB vaccines.

### 3.12. Esx5

The products of the Esx5 system activate the inflammasome pathway in the host cells, facilitating the death of the cells and escape of the *Mtb* [49,61]. This system comprises 17 genes, including five encoding PPE25, PE18, PPE26, PPE27, and PE19 proteins, all containing strong T cell epitopes showing cross-reactivity with other non-Esx PE/PPE proteins [63,169]. Deleting the five *ppe-pe* genes (from *ppe25* to *pe19*) of the *esx5* renders the *Mtb* attenuated for growth in immunocompetent mice [169]. Further, C57BL/6 mice immunized with an *Mtb* Δ*ppe2*5-*pe19* and challenged with H37Rv had a reduced bacterial load in the lungs and spleen compared to BCG-vaccinated mice [169], indicating moderately better protection. In contrast, mice and guinea pigs immunized with the Δ*esx5* strain, which has a deletion of 17 genes in the *esx5* locus (*Rv1782-Rv1798*), showed similar levels of protection to BCG-vaccinated mice exposed to a virulent HN878 strain of *Mtb* [170]. Better protection by the Δ*esx5* vaccine was noted only when administered using a prime-boost strategy with BCG as the prime vaccination [170]. Enhanced protection correlated with increased numbers of activated monocytes, central memory T cells (TCM), and follicular T cells (TFH) [170], although the mechanisms behind the increased protection by prime-boost vaccination remain unclear. In the same study, an *Mtb*Δ*esx-3* mutant, which has a deletion of 11 genes, was also tested, but its protection seems to be lower than that of the Δ*esx5* strain. The authors of this study opined that the large-scale deletion of genes in the Δ*esx5* vaccine could safeguard its potential reversion to virulence. However, this may lead to the loss of protective T cell epitopes in proteins encoded by the deleted genes. Since Δ*esx5* is highly attenuated in immunocompromised SCID mice [170], it has an interesting potential to be developed as a vaccine for HIV-infected children who are susceptible to TB.

### 3.13. UreC

Secreted urease C (Rv1850) of mycobacteria neutralizes the acidic environment of the phagosome of the macrophages, contributing to phagosomal maturation arrest [174,175]. Although BCG lacks the RD1 locus and is attenuated, it retains several genes related to phagosomal maturation arrest, including *ureC*. Consequently, BCG is sequestered within the neutral pH phagosome [176]. To nullify this effect, Kaufmann and his colleagues used a novel strategy of integrating the gene *hly*, which encodes the *Listeria monocytogenes* derived listeriolysin (LLO) toxin, into the chromosome of BCG, concurrently deleting *ureC* in its chromosome [177]. They proposed that BCG-secreted LLO could perforate the phagosomal membrane and allow the bacteria into the cytosol for bacterial antigen processing through the MHC-I pathway. The deletion of *ureC*, on the other hand, could favor the vATPase-mediated acidic pH required for LLO activity. They constructed two isogenic strains, BCG::*hly* and BCG::*hly*Δ*ureC*, and assessed their vaccine efficacy in BALB/c mice [177]. Both showed better protection over the parental strain, although protection by BCG::*hly*Δ*ureC* was superior [177]. This was due to the induction of apoptosis, increased antigen processing through MHC-I, the elicitation of T cells like TCM, TFH, and Th17, and high IgG antibody levels produced by BCG::*hly*Δ*ureC* [177,178]. Further, the vaccine had an excellent safety profile in mice, guinea pigs, newborn rabbits, and non-human primates [179]. Currently named VPM1002, it is one of the few live mycobacterial vaccines undergoing clinical trials in sub-Saharan Africa and India [14].

### 3.14. NuoG

This protein is one of the subunits of type-I NADH dehydrogenase of *Mtb* and BCG and was identified by screening for anti-apoptotic genes in *Mtb* [36]. *Mtb* uses NuoG (Rv3151) to inhibit host apoptosis by neutralizing the ROS derived from NOX2 [74]. Since the vaccine-induced apoptosis of macrophages increases vaccine efficacy [177], *nuoG* gene deletion in BCG and *BCG::hlyΔureC* was created to improve vaccine efficacy [101]. Both BCGΔ*nuoG* and BCG::*hly*Δ*ureC*Δ*nuoG* seem to enhance apoptosis in murine lymph nodes and improve autophagy activity in macrophages [101]. Consequently, both strains showed enhanced immunogenicity and efficacy against TB in mice challenged with *Mtb* H37Rv [101]. Specifically, the lung CFUs of BCGΔ*nuoG*- and BCG::*hl*yΔ*ureC*Δ*nuoG*-immunized mice demonstrated significant protection against TB. Protection against the more virulent *Mtb* Bejing W strain was also observed in mice immunized with BCG::*hly*Δ*ureC*Δ*nuoG* after 90 and 180 days post-challenge. The microarray analysis of draining lymph nodes from vaccinated mice showed an increased expression of genes related to GTPase activity, inflammatory responses, cell activation, and cell proliferation [101].

Additionally, the vaccine increased CD4+ TEM cells, TFH cells, germinal center B cells, and CD4+ TCM cells. Paradoxically, a *nuoG* mutant made in BCG China sub-strain had no significant protection in the *Mtb*-challenged mice [168]. These data suggest the possibility that the deletion of a gene in BCG sub-strains may give different effects, and this seems to be important because at least five major sub-strains of BCG (Copenhagen/Danish, Russian, Shanghai/China, Japan, Moreau) are used around the world for the primary immunization of infants.

**Table 1:** Efficacy of live *Mycobacterium tuberculosis* vaccines with gene(s) deleted for secreted protein(s).

Vaccine Name	Vaccine Components	Secreted Protein(s) Absent	Immunization Route/Dose	Challenge *Mtb* Strain	Challenge Route/Dose	Animal Model (Strain)	Efficacy in Relation to BCG	log_10_ CFU/LUNGS Reduction Than BCG	Ref.
**Δ*fbpA***	H37Rv strain with single gene (*fbpA*) knockout.	FbpA or Ag85A	SC/10^5^ CFU/mouse	Erdman	Aerosol/2.5 log_10_ CFU per mouse	Mouse (C57BL/6)	Better than BCG	~1.5	[115]
**Δ*glnA1***	H37Rv strain with single gene (*glnA1*) knockout.	Glutamine synthetase A1	SC/10^6^ CFU/mouse	Erdman	Aerosol/200 CFU per mouse	Mouse (C57BL/6)	Equal to BCG	-	[148]
**Δ*glnA1EA2***	H37Rv strain with 3 genes (*glnA1*, *glnE*, and *glnA2*) knockout.	Glutamine synthetase A, E and A2	SC/10^6^ CFU/mouse	Erdman	Aerosol/200 CFU per mouse	Mouse (C57BL/6)	Equal to BCG	-	[148]
**Δ*19***	H37Rv strain with single gene (*lpqH*) knockout.	Lipoprotein LpqH	SC/10^6^ CFU/mouse	H37Rv	Aerosol/100 CFU per mouse	Mouse (C57BL/6)	Equal to BCG	-	[130]
**Δ*mms***	H37Rv strain with 3 genes (*ptpA*, ptpB, and *sapM*) knockout.	Phosphatases PtpA, PtpB, and SapM	ID/5 × 10^5^ CFU/guinea pig	H37Rv	Aerosol/10–30 CFU per guinea pig	Guinea pigs	Better than BCG	0.83–4 weeks post-challenge; 1.41–12 weeks post-challenge	[154]
**Δ*bfrB***	H37Rv strain with single gene (*bfrB*) knockout	Bacterio-ferritin B	SC/10^6^ CFU/mouse	H37Rv	Aerosol/100 CFU per mouse	Mouse (C57BL/6)	Equal to BCG	-	[140]
**Δ*bioA***	H37Rv strain with single gene (*bioA*) knockout	BioA or 7,8-diaminopelargonic acid synthase	ID/10^6^ CFU/guinea pig (single or double dose with 6 week interval)	Erdman	Aerosol/50 CFU per guinea pig	Guinea pigs	Equal to BCG	-	[145]
**Δ*mmsb***	H37Rv strain with 4 genes (*ptpA, ptpB, sapM,* and *bioA*) knockout.	Phosphatases PtpA, PtpB, SapM and BioA	ID/5 × 10^5^ CFU/guinea pig	H37Rv	Aerosol/10–30 CFU per guinea pig	Guinea pigs	Less than BCG	-	[155]
***mc^2^6206*** **Δ*cpsA***	H37Rv strain with 3 genes (*leuD*, *panCD*, and *cpsA*) knockout.	CpsA	SC/10^6^ CFU/mouse	H37Rv	Aerosol/400 CFU per mouse	Mouse (C57BL/6)	Equal to BCG	-	[142]
**Δ*lprG***	H37Rv strain with two genes (*lprG* and *Rv1410c*) knockout.	Lipoprotein LprG	SC/~10^6^ CFU/mouse	H37Rv and Erdman	Aerosol/75 CFU per mouse or Aerosol/1 Median Infectious Dose (1MID50).	Mouse (C57BL/6, BALB/c and C3HeB/FeJ)	Equal or better than BCG	0.67–0.9 (in C3HeB/ FeJ mice);	[134,135]
***SO2***	MT103 strain with single gene (*phoP*) knockout.	All secreted proteins that are affected by PhoP	SC/10^7^ CFU/mouse	H37Rv	IV/2.5 × 10^5^ CFU per mouse	Mouse (BALB/c)	Equal to BCG	-	[180,181]
			SC/5 × 10^4^ CFU/ guinea pig	H37Rv	Aerosol/10–50 CFU or 500 CFU per guinea pig	Guinea pigs (Dunkin Hartley)	Better than BCG in high-dose challenge	>1	
			ID/5 × 10^5^ CFU/macaques	Erdman	IT/1000 CFU per macaques	Rhesus macaques (Macaca mulatta)	Better than BCG	0.77	
**Δ*ppe25-pe19***	H37Rv strain with 5 genes (*ppe25*, *pe18*, *ppe26*, *ppe27,* and *pe19*) knock out.	PPE25, PE18, PPE26, PPE27 and PE19	SC/10^6^ CFU/mouse	H37Rv	Aerosol/100 CFU per mouse	Mouse (C57BL/6)	Better than BCG	~0.5	[169]
**Δ*secA2***	mc^2^3112 strain with single gene (*secA2*) knock out.		SC/10^6^ CFU/mouse	Beijing/W (HN878) or Erdman	Aerosol/50–100 CFU per mouse	Mouse (C57BL/6)	Better than BCG	0.72	[182]
			ID/10^3^ CFU/guinea pig	H37Rv	Aerosol/10–30 CFU per guinea pig	Guinea pigs (Dunkin Hartley)	Better than BCG in lymph node but not in lungs	-	
**Δ*secA2*Δ*lysA***	mc^2^3112 strain with double gene (*secA2* and *lysA*) knockout.	Proteins secreted by SecA2 secretion system	SC/10^6^ CFU/mouse	Erdman	Aerosol/50–100 CFU per mouse	Mouse (C57BL/6)	Better than BCG	0.66	[183]
***MTBVAC***	MT103 strain with double gene (*phoP* and *fadD26*) knockout.	All secreted proteins affected by PhoP	SC/5 × 10^5^ CFU/mouse	H37Rv	IN/100 CFU per mouse	Mouse (C57BL/6)	Better than BCG	~0.5	[184,185]
			SC/5 × 10^3^ − 5 × 10^5^ − CFU/guinea pig	H37Rv	Aerosol/10–50 CFU per guinea pig	Guinea pigs (Dunkin Hartley)	Equal to BCG	-	
			ID/8.2 × 10^5^ CFU/macaques	Erdman	Aerosol/14–30 CFU per macaques	Rhesus macaques (Macaca mulatta)	Better than BCG but not in CFU	-	
***MTB*** **VAC erp^-^**	MT103 strain with triple gene (*phoP*, *fadD26,* and *erp*) knock out.	All secreted proteins which are affected by PhoP and Erp	ID/10^5^ CFU/mouse	H37Rv	IT/10^3^ CFU per mouse	Mouse (C57BL/6)	Equal to BCG	-	[186]
**Δ*esx-5***	H37Rv strain with 17 genes (*eccB5*, *eccc5*, *cyp143*, *Rv1786*, *ppe25*, *pe18*, *ppe26*, *ppe27*, *pe19*, *esxM*, *esxN*, *ncRv11793*, *Rv1794*, *eccD5*, *mycP5*, *eccE5,* and *eccA5*) knock out.	ECCB5, ECCC5, CYP143, RV1786, PPE25, PE18, PPE26, PPE27, PE19, ESXM, ESXN, NCRV11793, RV1794, ECCD5, MYCP5, ECCE5, and ECCA5	IM/10^6^ CFU/mouse (2 dose with 6 week interval)	HN878, and H37Rv	Aerosol/40–100 CFU per mouse	Mouse (C57BL/6)	Equal to BCG	-	[170]
			IM/10^4^ CFU/guinea pig	Beijing 212	Aerosol/10–20 CFU per guinea pig	Guinea pigs (Dunkin Hartley)	Equal to BCG	-	
**Δ*esx-3***	H37Rv strain with 11 genes (*eccA3*, *eccB3*, *eccC3*, *pe5*, *ppe4*, *esxG*, *esxH*, *espG3*, *eccD3*, *mycP3*, and *eccE3*) knock out	ECCA3, ECCB3, ECCC3, PE5, PPE4, ESXG, ESXH, ESPG3, ECCD3, MYCP3, and ECCE3	IM /10^4^ CFU/guinea pig	Beijing 212	Aerosol/10–20 CFU per guinea pig	Guinea pigs (Dunkin Hartley)	Not mentioned	-	[170]

Note: subcutaneous (SC); intradermal (ID); intramuscular (IM); intravenous (IV); intranasal (IN); intratracheal (IT). Efficacy was determined based on lung CFU load in comparison with the wild-type BCG strain at least at one-time point. Log_10_ CFU was not mentioned for some of the studies for which we stated the approximate values based on bar graphs.

### 3.15. SecA2

As discussed above, *Mtb* possesses two secretory transport systems: the primary SecA1 (Rv3241c) and the accessory SecA2 (Rv1821). The latter is predicted to be a unique system in *Mtb* to transport a small subset of *Mtb* proteins related to its pathogenicity. Consistent with this prediction, an *secA2* deletion mutant in *Mtb* H37Rv revealed its role in pathogenicity [187]. An *secA2* mutant showed diminished SodA expression and relatively attenuated growth in immunocompetent and SCID mice and in mouse macrophages derived from Phox(−/−) and Nos2(−/−) mice [188]. Further, macrophages infected with this strain induced relatively higher levels of TNF-α, IL-2, IFN-γ, and reactive nitrogen intermediates (RNI) than macrophages infected with *Mtb* H37Rv, suggesting an immunosuppressive role for SecA2 [188].

Interestingly, the *secA2* mutant enhanced apoptosis in macrophages and the priming of antigen-specific CD8+ T cells in mice [182], which led to its evaluation as a vaccine in mice and guinea pigs. C57BL/6 mice and guinea pigs immunized with a Δ*secA2* strain and challenged with HN878 and H37Rv showed a better reduction in CFUs against TB compared to BCG [182]. The lung CFUs of Δ*secA2*-immunized mice were 0.72 log^10^ lower than those lung CFUs of mice vaccinated with BCG. This reduction in CFUs was also accompanied by a decrease in histopathological scores for the lungs in Δ*secA2*-immunized mice [182]. Interestingly, it was proposed that Δ*secA2* mutant effects were due to SodA, the only enzyme affected by *secA2* deletion at that time. However, current literature reveals that SecA2 is responsible for transporting several host effector proteins released by *Mtb*, including SapM, Ndk, PknG, LdpC, and others [46]. Thus, *secA2* deletion is likely to decrease the expression of multiple proteins.

Nonetheless, an *Mtb* mutant lacking both SecA2 and LysA proteins showed increased protection against TB in mice compared to BCG [183]. Despite the efficacy of the Δ*secA2*/Δ*lysA* mutant, it has not advanced as a vaccine candidate, which is slightl surprising. In addition, *secA2* deletion in BCG had no impact on its immunogenicity [183], suggesting that *secA2* may differently affect the genes of *Mtb* and BCG.

### 3.16. PhoP

PhoP is a sensor component of the PhoP–PhoR two-component regulatory system and an important virulence factor [189], regulating approximately 2% of the genes in the *Mtb* genome [190]. The loss of virulence in *Mtb* H37Ra (avirulent strain) is partly related to a point mutation in the *phoP* gene (*Rv0757*) [191]. Although PhoP is not a secretory protein, evidence indicates that it indirectly controls the translocation of the secreted proteins ESAT-6 and CFP-10 of the ESX1 secretion system [192]. It appears that the translocation of ESAT-6 and CFP-10 to the bacterium’s surface requires the products of *espACD* genes located within the ESX1 system, whose expression is controlled by *phoP* [193]. Thus, the deletion of the *phoP* gene could affect the expression of the *espACD* genes and, consequently, the translocation of ESAT-6 and CFP-10 [194,195]. The absence of *phoP* results in the secretion of CFP-10 independently of ESAT-6, eliciting immune responses to CFP-10 in both mice and non-human primates upon MTBVAC exposure [184,196]. A Δ*phoP* vaccine *SO2* developed from a clinical strain of *Mtb* MT103 belongs to this category [180]. The *SO2* vaccine was severely attenuated in SCID mice and conferred superior protection against TB compared to BCG in mice, guinea pigs, and non-human primates [180]. Later, to meet the stipulations of the Geneva Consensus [197], the *SO2* vaccine was genetically modified as a marker-less double mutant with deletions in *phoP* and *fadD26*, and this new construct was named the MTBVAC vaccine [185]. Compared to BCG, MTBVAC showed enhanced safety, increased immunogenicity, and efficacy in animal models such as mice, guinea pigs, and non-human primates [184,185,196,198]. The MTBVAC vaccine protects macaques better than BCG by reducing disease pathology measured by in vivo imaging using CT scans, macroscopic pathological lesions examined at necropsy, and studying the frequency and severity of pulmonary granulomas [184]. The immune signatures after MTBVAC vaccination also included higher levels of Th1 cytokines response, especially poly- (IFN-γ+TNF-α+IL2+) and multi-(IFN-γ+TNF-α+) functional CD4+ T cells. After rigorous preclinical studies, the MTBVAC vaccine entered a clinical trial in Africa [199]. MTBVAC is an example of a vaccine developed through the rational deletion of genes in *Mtb* for eventual human application.

### 3.17. Mpt

Two homologous secreted proteins, namely Mpt70 (Rv2875) and Mpt 83 (Rv2873), were found to be highly immunogenic in humans and mice [200]. The genes encoding these proteins are conserved in both *Mtb* and BCG. A recent study evaluated a mutant in which these two genes and *esxS*, *espC*, and *espA* of the ESX system in BCG create a five-gene knockout Δ*BCG-TK* [201]. Interestingly, Δ*BCG-TK* showed vaccine efficacy similar to that of wild-type BCG in mice, and one can propose a contradictory observation that knocking out genes encoding secretory proteins may not affect vaccine efficacy. However, as noted above, with Δ*lprG* and Δ*cpsA* mutants, the genetic background of BCG or *Mtb* used to create a mutant may decide the vaccine’s immunogenicity.

### 3.18. Erp

*Mtb* encodes a 28 kDa secretory protein named exported repeated protein (Erp; Rv3810) that contains 11 proline–glycine–leucine–threonine–serine (PGLTS) repeats [202]. Mycobacterial *erp* mutants are attenuated in macrophages, zebrafish embryos, mice, and leopard frogs [203,204]. Erp is found to interact with another gene called *Rv2212*, an adenylyl cyclase. Through this interaction, Erp enhances Rv2212-mediated cyclic AMP (cAMP) production, which seems to lower the intracellular survival of *Mtb* Δ*erp*. We note here that the deletion of the *erp* gene in the MTBVAC strain (MTBVAC *erp* (-) further attenuated its growth in SCID mice compared to MTBVAC and BCG. Although MTBVACΔ*erp* generates protection similar to BCG, because of its higher safety profile, MTBVAC *erp* (-) has been recommended for the vaccination of immune-suppressed populations, such as people with HIV, where BCG causes disseminated disease, also known as BCGosis [186].

### 3.19. BCG_1419c

BCG_1419c is a cyclic dimeric GMP (c-di-GMP) phosphodiesterase (PDE) protein with phosphodiesterase activity [205]. This protein is encoded by gene *BCG_1419c* in the BCG Pasteur strain and by the gene *Rv1357c* in *Mtb* H37Rv and it was reported to degrade bis-(30–50)-c-di-GMP, which is linked to biofilm formation and virulence [205]. BCG_1419c is not a secretory protein. However, similar to the *phoP* mutant, which affects the secretion of ESAT-6 and CFP10, the BCG∆*BCG1419c* mutant vaccine increases the expression of secreted proteins like Tuf, GroEL1, DnaK, and GroES, while showing reduced levels of GroEL2 and AhcY/SahH [206]. Remarkably, the BCGΔ*BCG1419c* vaccine strain demonstrates a better control of both active and chronic TB in murine and guinea pig models compared to saline control [207,208,209,210,211,212,213]. However, it does not provide better protection for animals in terms of reducing lung CFU compared to BCG. Interestingly, in chronic type 2 diabetes (T2D), murine model BCGΔ*BCG1419c* effectively reduces pneumonia in comparison to BCG-vaccinated mice [213].

**Table 2:** Efficacy of live BCG vaccines with gene(s) deleted for secreted protein(s).

Vaccine Name	Vaccine Components	Secreted Protein(s) Absent	Immunization Route/Dose	Challenge *Mtb* Strain	Challenge Route/Dose	Animal Model (Strain)	Efficacy in Relation to BCG	log_10_ CFU/LUNGS Reduction Than BCG	Ref.
**VPM1002** ***(*** **Δ*ureC::hly)***	BCG Pasteur strain with single gene (*ureC*) knockout, which expresses listeriolysin (*hly*).	Urease C	IV/10^6^ CFU/mouse	H37Rv or Beijing/W	Aerosol/30 or 200 CFU per mouse	Mouse (BALB/c)	Better than BCG	~0.5–2	[177]
***sapM::T***	BCG 1721 strain with single gene (*sapM*) knockout.	SapM phosphatase	SC/10^5^ CFU/mouse	H37Rv	IV/5 × 10^4^ CFU per mouse (or) IT/ 2 × 10^5^ CFU per mouse	Mouse (BALB/c)	Better than BCG	~0.5 (Luminescence)	[156]
**Δ*zmp1***	BCG Pasteur or Denmark strain with single gene (*zmp1*) knockout.	Zmp1 or Zinc containing metalloprotease 1	SC/5 × 10^4^ CFU/ guinea pig	H37Rv	Aerosol/10–50 CFU per guinea pig	Guinea pigs (Dunkin Hartley)	Better than BCG	~0.91	[164]
***BCG:*** **Δ*85B***	BCG Pasteur strain with single gene (*fbpB*) knockout.	FbpB/Ag85B	SC/5 × 10^5^ CFU/mouse	H37Rv	Aerosol/100 CFU per mouse	Mouse (C57BL/6)	Equal to BCG	-	[118]
**Δ*nuoG***	BCG Pasteur strain with single gene (*nuoG*) knockout.	NuoG type-I NADH dehydrogenase subunit G	SC/10^6^ CFU/mouse	H37Rv	Aerosol/100–200 CFU per mouse	Mouse (C57BL/6)	Better than BCG	~0.5	[101]
**Δ*ureC::hly* Δ*nuoG***	BCG Pasteur strain with double gene (*ureC*, *nuoG*) knockout, which expresses listeriolysin.	UreC and NuoG	SC/10^6^ CFU/mouse	H37Rv or Beijing/W	Aerosol/100–200 CFU per mouse	Mouse (C57BL/6)	Better than BCG	~0.8–2	[101]
**ΔBCG TK (triple knock-out)**	BCG Danish strain with five gene (*esxS*, *mpt70*, *mpt83*, *espC* and *espA*) knockout	EsxS, Mpt70, Mpt83, EspC, and EspA	SC/5 × 10^4^ CFU/guinea pig	*M. bovis* AF2122/97	Aerosol/10–20 CFU per guinea pig	Guinea pigs (Dunkin Hartley)	Equal to BCG	-	[201]
**Δ*BCG2432c***	BCG China strain with *eis* gene (*BCG2432c*) knockout	EIS or Enhanced Intracellular Survival protein	SC/10^6^ CFU/mouse	H37Rv	IN/100 CFU per mouse	Mouse (C57BL/6)	Better than BCG	~1–2	[168]
**Δ*BCG3174***	BCG China strain with *nuoG* gene knockout (BCG3174)	NuoG	SC/10^6^ CFU/mouse	H37Rv	Intranasal/100 CFU per mouse	Mouse (C57BL/6)	Equal to BCG	-	[168]
**Δ*BCG 1419c***	BCG Pasteur strain with single gene (*c-di-GMP phosphodiesterase*) knockout.	All secreted proteins affected by (*c-di-GMP phosphodiesterase*).	SC/8 × 10^3^ or 2.5 × 10^2^ or 5 × 10^4^ or 10^6^ or ~10^5^ or 10^7^ CFU/mouse	H37Rv or M2 or HN878	IT/2.5 × 10^5^ or 10^3^ or ~170 CFU per mouse or Aerosol/100–200 CFU per mouse.	Mouse (BALB/c, B6D2F1, C57BL/6, I/StSnEgYCit)	Equal to BCG or Better than BCG in chronic infection model	~0.8 (chronic infection model)	[207,208,209,210,211,212,213]
			ID/10^3^ CFU per guinea pig	H37Rv	Aerosol/10–20 CFU per guinea pig	Guinea pigs	Equal to BCG	-	

Note: subcutaneous (SC); intradermal (ID); intramuscular (IM); intravenous (IV); intranasal (IN); intratracheal (IT). Efficacy was determined based on lung CFU load in comparison with the wild-type BCG strain at least at one time point. Log_10_ CFU was not mentioned for some of the studies for which we stated the approximate values based on bar graphs.

## 4. Status of Mycobacterial Vaccines Deficient in Secreted Protein(s)

It is apparent from these studies that BCG is a ‘natural’ vaccine arising out of the deletion of genes encoding secretory proteins, as it lacks the Region of Difference 1 (RD1) that encodes several secretory proteins, including the immunodominant antigens ESAT-6 and CFP-10 [54]. Studies discussed above specifically targeted genes that played a role during pathogenesis for developing vaccines. Surprisingly, only a few *Mtb*-derived vaccines showed higher efficacy than BCG, and most were comparable to BCG. In contrast, many BCG mutants with deletions of secretory genes showed increased efficacy compared to the wild type. We recall here that *Mtb*- or BCG-derived vaccines that showed higher efficacy than BCG had deletions in *fpbA*, *sapM*, *zmp1*, *ureC*, *nuoG*, *secA2*, and *eis* genes. Intriguingly, except for the *fbpA*-encoded product, other genes modulated PL fusion, autophagy, apoptosis, and inflammasomes in the antigen-presenting cells (APCs) [34,35,36,61,83,214]. Although the disruption of *fbpA* in *Mtb* also led to increased PL fusion in APCs, the underlying mechanism remains unclear [215].

Because PL fusion, autophagy, apoptosis, and inflammasome activation ensure the efficient processing and presentation of vaccine antigens to the T cells by the APCs [216,217,218], it is apparent that the deletion of these genes enhanced the efficacy of the mutants against tuberculosis, justifying the strategy of secretory protein gene knockout. However, we need to recognize the caveat that these deletions of proteins that contain potent T and B cell epitopes like ESAT6 and CFP10 may lead to reduced immunogenicity. In addition, an in vitro phenotype may not always lead to increased immunogenicity. An example is *Mtb* lacking *cpsA*; although *Mtb*Δ*cpsA* enhanced autophagy in APCs, an increase in vaccine efficacy was not observed [142]. In this regard, deletion strategies should focus on those that interfere with PL fusion and autophagy (SapM, Zmp, PtpA, and PtpB).

It also appears that gene knockouts in BCG and *Mtb* will likely have different consequences because of the genetic background of the attenuated vaccine vs. virulent pathogen. Because BCG has an excellent safety record, the deletion of *sapM*, *zmp1*, and *eis* is likely to improve immunogenicity, since BCG contains multiple immunogenic genes, an Antigen85 complex, and others listed above. Intriguingly, *eis* deletion in BCG markedly reduced the *Mtb* load in the lungs of nasally challenged mice [168]. Further, the double deletion of *ureC* and *nuoG* synergistically enhanced the efficacy of BCG [101], suggesting that highly effective BCG mutants can be produced through multiple deletions of genes selected based on their functions. Herein, we again emphasize the need to exercise caution in selecting the parent platform, since the Δ*nuoG* BCG Pasteur strain was effective against TB [101] but not the Δ*nuoG* BCG China sub-strain [168].

Similar to BCG vaccine manipulations, using the *Mtb* platform to derive vaccines deleting secretory products appears encouraging. The Δ*secA*-Δ*lysA* and Δ*fbpA*-*sapM* and mutants were more effective than BCG [116,152,183] and are potential booster vaccines for BCG-vaccinated infants. Among these, the Δ*secA*-*lysA* strain has an excellent safety profile in mice [183] compared to Δ*fbpA*-*sapM*, qualifying it for clinical trials. Our unpublished observations indicate that the safety profile of the Δ*fbpA*-*sapM* mutant in mice is lower than that of BCG and may need additional gene deletions. Although Δ*lprG* derived from *Mtb* also seems to be a strong candidate vaccine, its superior efficacy over BCG is apparent only in TB-susceptible C3HeB/FeJ mice but not in the TB-resistant C57/BL6 mice [134].

## 5. Future Directions and Conclusions

A significant impediment to developing vaccines against TB is the lack of reliable immune correlates of protection [219,220,221,222]. Studies with mice, guinea pigs, and rabbits revealed that CD4+ T cells secreting IFN-γ, TNF-γ, and IL-1β and CD8+ T cells secreting granulysin and perforin play a critical role in defending *Mtb* infection [223,224]. Recent studies show that TH17 T cells secreting IL-17 are also a protective parameter [225].

All new TB vaccines aim to enhance T cell-dependent immune responses in the host. Interestingly, the role of innate immunity in designing more efficacious vaccines has received less attention [226]. An intriguing example is the induction of trained immunity by the BCG vaccine, which activates dectin signaling, generating protection against TB through epigenetic modifications of macrophages, neutrophils, and DCs [227]. Although BCG-induced trained immunity seems more effective against nontuberculous infections, it remains unclear to what extent trained immunity affects adaptive immunity during tuberculosis vaccination. It has been reported that BCG-induced protection wanes by year 5 in children, and TB continues to occur despite vaccination [228].

In this context, we noted that deleting multiple secretory proteins led to enhanced autophagy and inflammasome activation that delivered vaccines to lysosomes for better immune responses (our unpublished work). Autophagy and inflammasome pathways are major innate immunity pathways triggered by multiple mechanisms of mycobacteria, including TLR, NLR, and C-type lectin (also known as dectins) signaling. Intriguingly, dectin-dependent trained immunity induced by BCG cell wall components is regulated by autophagy [229]. There is, therefore, a pressing need to investigate whether new-generation tuberculosis vaccines can be genetically manipulated to activate both innate (autophagy and inflammasome) and adaptive T cell-dependent arms of immunity. For example, *Mtb* was reported to secrete a lysine acetyltransferase that epigenetically modified the ability of macrophages to secrete anti-inflammatory cytokines like IL-10.

Moreover, the acetylation of histones associated with the genes regulating autophagy regulates the induction of autophagy [230]. A lysine acetyltransferase mutant of *Mtb* may induce robust autophagy and show better protection. Further, *Mtb*-derived methyl transferases hyermethylate the DNA of tuberculosis patients, reducing their immune responses [231]. We propose that deletion mutants of *Mtb* that lack acetylase, methylase, or both may serve as promising vaccine candidates.

An attractive alternative approach to enhance innate immunity is to integrate ‘adjuvant’ active molecules such as TLR agonists into candidate vaccines to overcome immune-suppressing proteins. We recently fused a TLR2-activating CFP-10-derived peptide C5 with Ag85B and expressed it in BCG through a plasmid. The recombinant BCG85BC5 vaccine enhanced protection against TB and induced significant levels of T-effector and T-central memory cell response in mice [108]. We propose that integrating adjuvant constructs like this into Δ*sapM* or Δ*zmp1* mutants will markedly improve protection associated with long-term memory.

Finally, recent reports indicate that the humoral immune response against *Mtb* may also play a significant role in protection against TB [101,184]. In this direction, we propose that deletion mutants can be made to express antibody-inducing peptide epitopes.

## Data Availability

Not applicable.
